# A Case Report of Asherman’s Syndrome With Abnormal Placenta Implantation (Intrauterine Adhesion)

**DOI:** 10.7759/cureus.39878

**Published:** 2023-06-02

**Authors:** Laith A Ayasa, Jasmin O Abdallah, Motaz Saifi, Ahmed Wafi

**Affiliations:** 1 Internal Medicine, Al-Quds University, Jerusalem, PSE; 2 Medicine, An-Najah National University, Nablus, PSE; 3 Gynecology, Clinique Anne St Remi, Bruxelles, BEL

**Keywords:** abnormal placentation, prenatal diagnosis, magnetic resonance imaging (mri), 3-d ultrasound, intrauterine adhesion

## Abstract

We report the case of a 28-year-old patient with a partial placental insertion on an intrauterine adhesion diagnosed at 20 weeks’ gestation. The increasing incidence of intrauterine adhesions during the last decade has been attributed to the rising number of uterine surgeries in the fertile population and better imaging studies facilitating diagnosis. Although uterine adhesions during pregnancy are generally considered benign, the existing evidence is conflicting. The obstetric risks in these patients are unclear, but higher numbers of placental abruption, preterm premature rupture of membranes (PPROM), and cord prolapse have been reported. Thus, a prenatal diagnosis should prompt close feto-maternal observation. Surgical resection should be offered to patients with adhesions found prior to pregnancy.

## Introduction

Mahony et al. first defined the amniotic sheet as a septation within the amniotic cavity that is believed to result from intrauterine adhesions (IUA) [1]. Although the exact etiology of IUA is unknown, it is likely that any intrauterine surgery causing injury to the basilar layer of the endometrium can contribute to their formation [2]. The maternal origin of an amniotic sheet is suggested by its close association with a maternal history of curettages, cesarean deliveries, and endometritis [3]. It would be worth mentioning that IUAs are observed in 0.14-0.60% of pregnant women, and they were estimated to occur in one out of every five women following a miscarriage, adding that they have had an increased incidence in the recent decade [4]. During pregnancy, as the gestational sac expands and drapes over the adhesion, an amniotic sheet develops, partially dividing the gestational sac [5]. Although generally deemed benign, the presence of an amniotic sheet raises the risk of developing placental complications, including placental abruption and abnormal placentation, which were reported in 26.1% of such cases [5,6]. The diagnosis is made using Color Doppler, which detects the presence of maternal blood vessels inside the sheet, and it can be further confirmed with pathological reports [5]. Herein, we report a case of partial placental insertion on an amniotic sheet in the center of the uterine cavity that was managed successfully.

## Case presentation

A 28-year-old woman (gravida 5, para 1), with a past surgical history of one cesarean section and three first trimester dilation and curettage (D&C) procedures, was found to have uterine abnormalities at 20 weeks' gestation. Ultrasonography revealed the presence of a broad intrauterine structure with a horizontal plane connecting the anterior and posterior uterine walls. Doppler ultrasonography demonstrated blood flow within the amniotic sheet, raising concern for an amniotic sheet with abnormal placental insertion.

**Figure 1:**
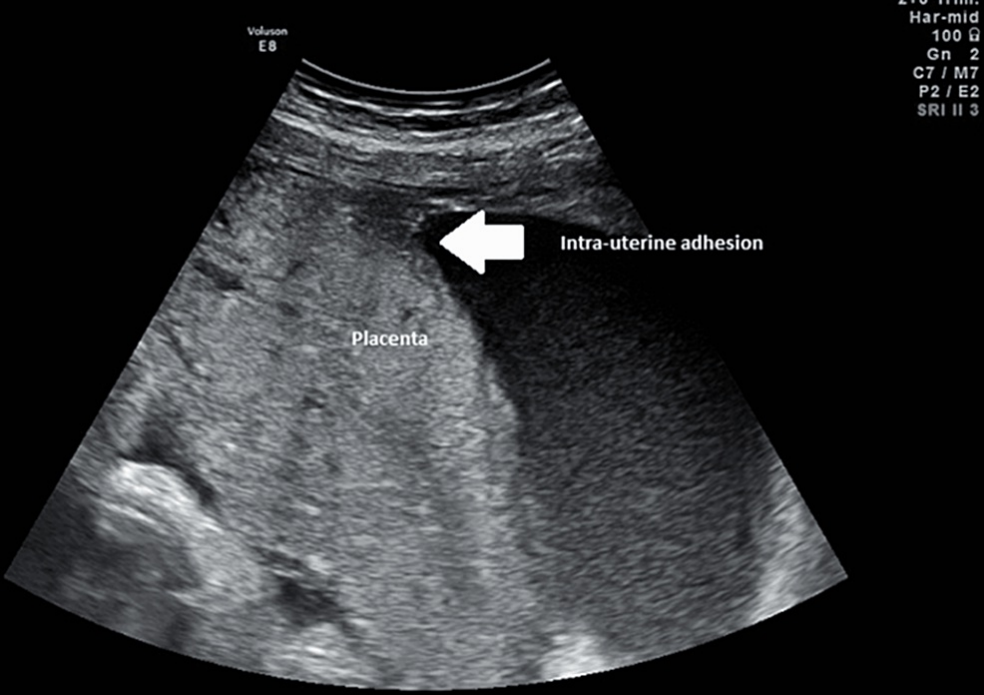
Transabdominal ultrasonography displaying placental implantation on the adhesion (white arrow) and on the posterior uterine wall.

At 26 weeks’ gestation, ultrasonography showed an abnormally implanted placenta on the adhesion and on the posterior uterine wall. It also showed a velamentous insertion of the umbilical cord close to the intrauterine adhesion (Figure 1). The measured fetal growth parameters, fetal anatomy scan, and umbilical artery Doppler flow were all within the normal range, thus eliminating the possibility of amniotic band syndrome.

**Figure 2:**
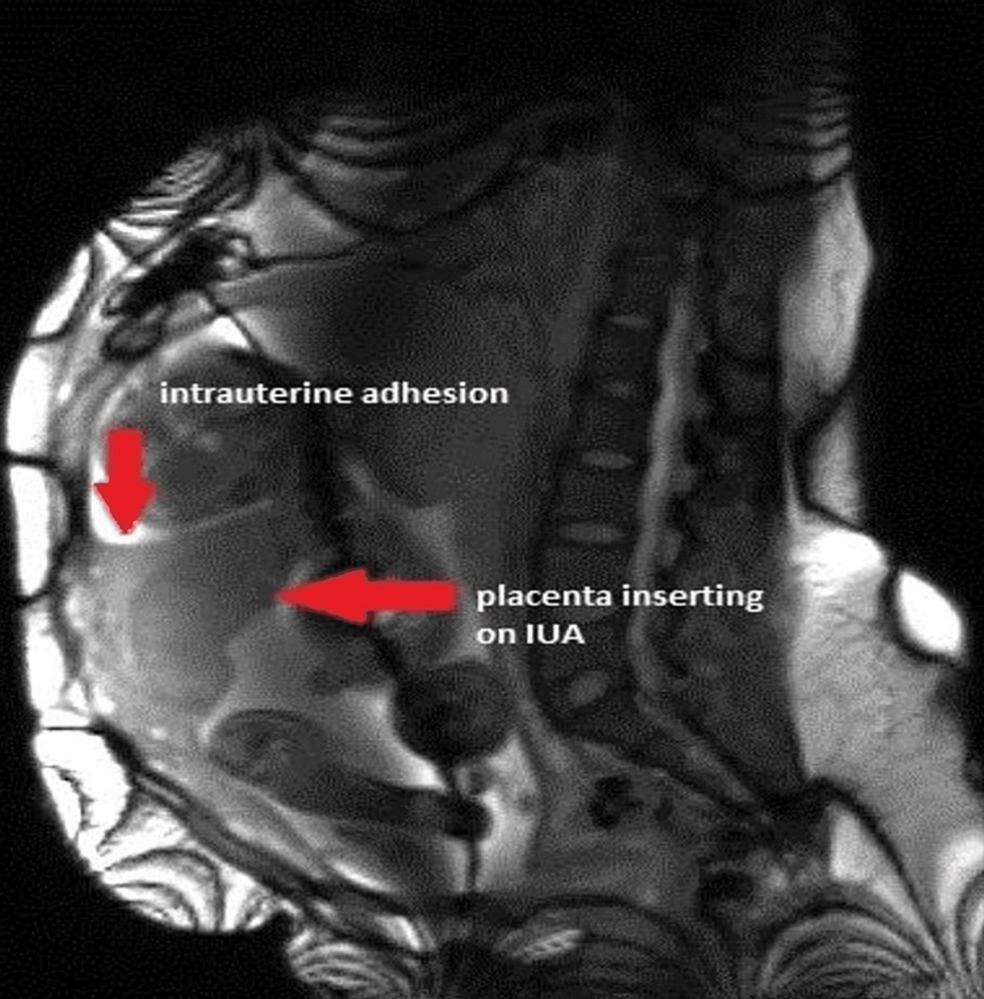
Sagittal MRI displaying an antero-posterior adhesion (vertical red arrow) and placental insertion covering two-thirds of the adhesion (horizontal red arrow). MRI, magnetic resonance imaging

The aforementioned findings were later confirmed by a magnetic resonance imaging (MRI) scan done at 30 weeks and 6 days of gestation. These findings included the anteroposterior adhesion (Figure 2), which now followed an upward course from the lower segment toward the fundus of the uterus, as well as the placenta covering two-thirds of the adhesion, in addition to the velamentous insertion of the umbilical cord on the adhesion.

At the following ultrasound examination conducted at 33 weeks’ gestation, an increased tension on the intrauterine adhesion was observed, raising concern for its disruption with the risk of placental abruption and fetal demise. The patient was admitted for close fetal surveillance and administration of dexamethasone to assure fetal lung maturity.

At 34 weeks’ gestation, an elective cesarean section was performed under epidural anesthesia. A female infant weighing 2,400 g was delivered with an APGAR score of 4 and 7 at 1 and 5 minutes of life, respectively; the neonate was admitted to the neonatal intensive care unit because of prematurity. The placental weight was 490 g.

**Figure 3:**
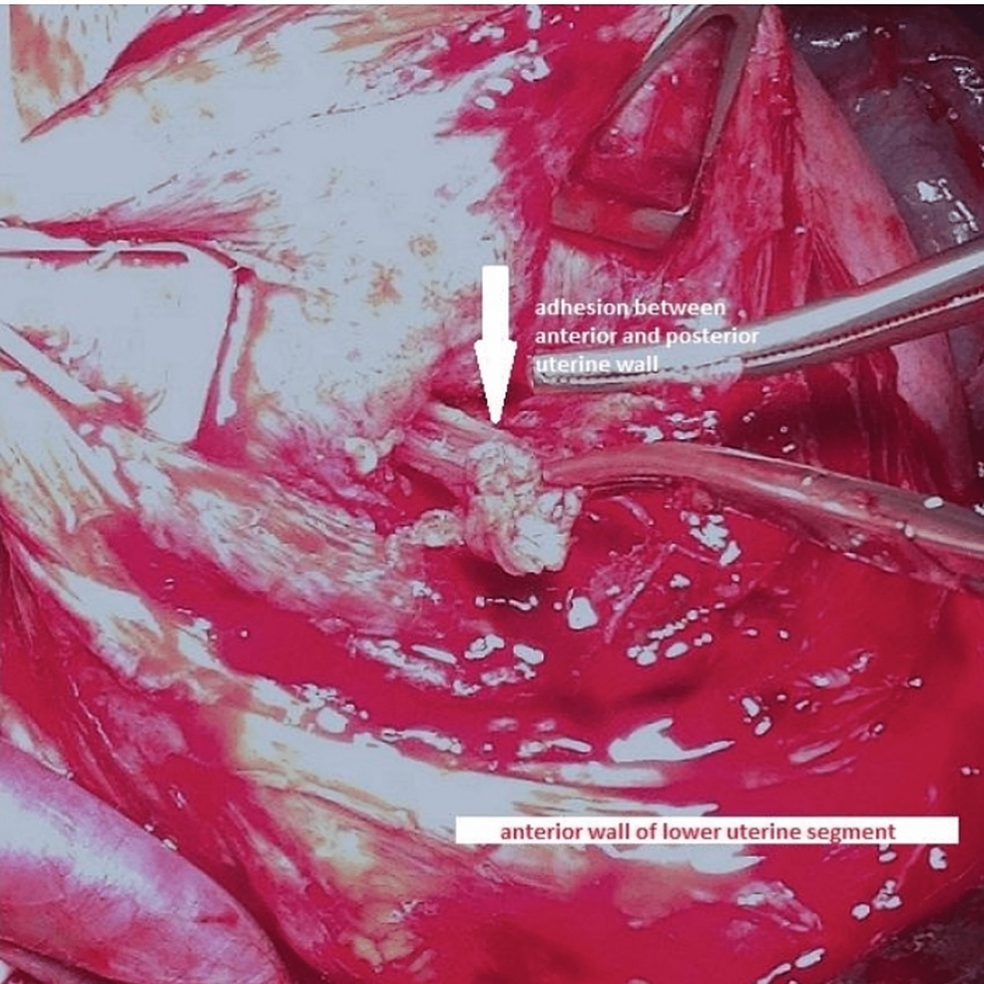
Intraoperative picture displaying fine adhesions connecting the posterior and the anterior wall (white arrow).

**Figure 4:**
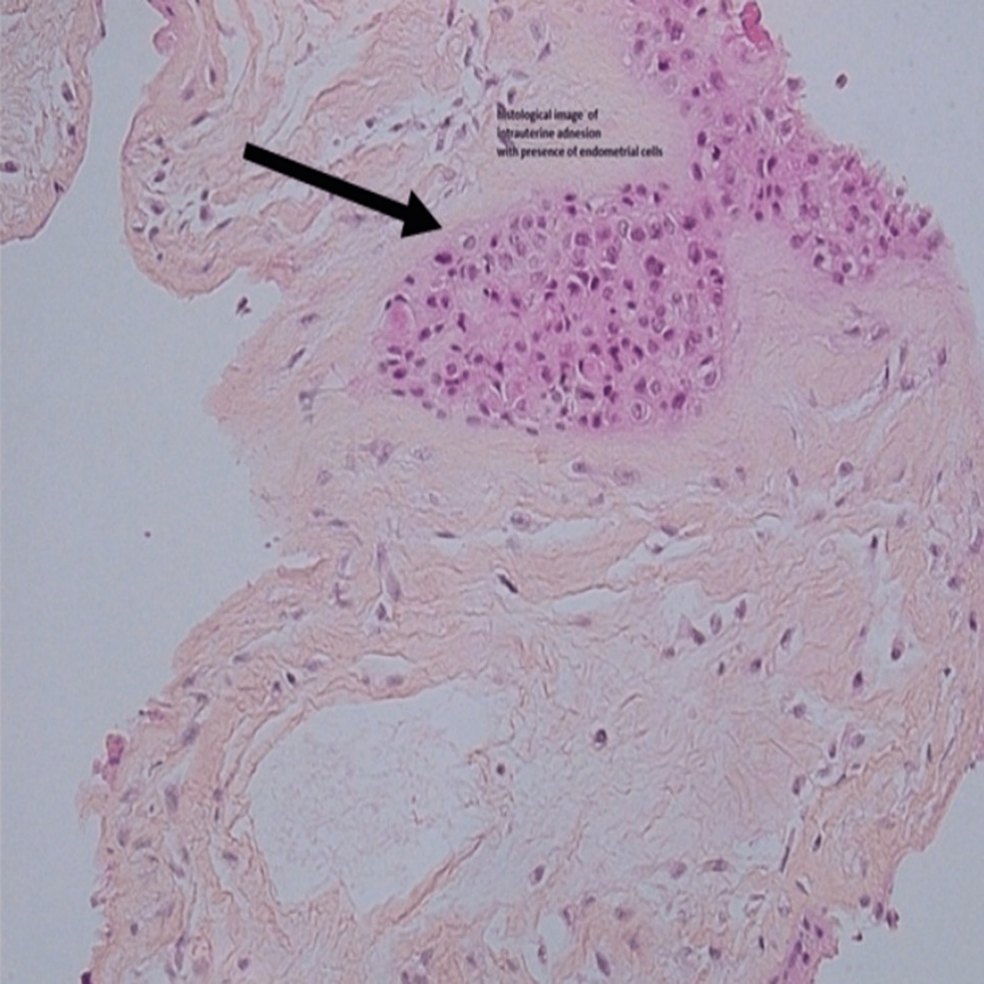
Pathological examination of the removed adhesion shows myometrial and endometrial tissue (black arrow).

During the cesarean section, the placenta was found partly adherent over the adhesion. Additionally, visual inspection revealed multiple fine adhesions connecting the posterior and anterior walls (Figure 3). No other uterine abnormalities were noted. Pathological examination after resection confirmed the diagnosis of an adhesion containing myometrial and endometrial tissue (Figure 4). An intrauterine copper device was inserted at the end of the intervention to reduce the risk of recurrence. The maternal course and neonatal development were uneventful and had favorable clinical courses.

## Discussion

Asherman's syndrome (AS) is characterized by the occurrence of adhesions within the endocervix and the intrauterine location, which can eventually lead to potential complications such as amenorrhea, decreased fertility, increased risk of pregnancy loss, and abnormal placental implantation [7]. It is reported that over 90% of AS cases are related to D&Cs performed after pregnancy, which is commonly carried out to remove any retained products following delivery or abortion [8]. Various other factors can potentially contribute to the formation of intrauterine adhesions, such as infections, intrauterine instrumentations, and surgical interventions like myectomy, in addition to ablation and hysteroscopic procedures [9]. The subsequent damage to the endometrium can result in abnormalities in the basal decidua of the endometrium (Nitabuch's layer), compromising the integrity of the intrauterine cavity and resulting in its obliteration and abnormal placentation [10].

A retrospective cohort study conducted by Feng et al. to investigate the outcomes that may be seen in third-trimester women who underwent hysteroscopic adhesiolysis previously reported an incidence of placental complications, with retained placenta having the highest incidence (42%), abnormally invasive placenta (34%), and placenta previa (12%) being the least [11]. One of the possible manifestations of AS is the formation of an amniotic sheet, which would appear as a delicate “Y-shaped” structure that spans across the uterine cavity resulting from the reflection of a part of the chorioamnion over an intrauterine adhesion, mimicking the intertwin membrane in a dichorionic diamniotic twin pregnancy [12]. Tan et al. described two types of amniotic sheets on ultrasound: (1) a complete sheet leading to complete transverse separation of the amniotic sac and (2) an incomplete sheet with a free-floating edge [13]. On Doppler ultrasonography, amniotic sheets are characterized by the presence of maternal blood flowing through them [14]. Despite the abovementioned studies, the number of published articles concerning the obstetric complications of IUA is limited, and more large observational studies should be conducted.

Amniotic sheets can disrupt normal placental positioning, where a portion of them would get implanted on the adhesions, compromising sufficient perfusion (blood flow) to the affected area, which can result in various placental complications and possibly fetal death [5]. For instance, preterm premature rupture of membrane (PPROM) appears to be two times more prevalent in pregnancies complicated by IUA (5.5%-6.6%) compared to 0.4%-1% of the general population [15]. Preterm labor and birth are increased in these pregnancies too, given that up to 18% are born prior to 37 weeks’ gestation, with 3.4%-11.5% being delivered prior to 34 weeks’ gestation [4,6].

Placental abruption is another noteworthy complication with a reported two- to eight-fold increased risk of development (2.1% (Tuuli) to 3.3% (Nelson)) [4,6]. The authors hypothesized that placental implantation next to or on the adhesions increases the risk of placental abruption due to decreased vascularization and the progressively increasing tension on the adhesions as pregnancy advances. In their retrospective study of 44 cases, Tan et al. reported an association between the localization of the IUA (especially in the lower segment) and pregnancy-associated complications. Also, Tan reported two cases of intrauterine fetal death (IUFD); in one of these cases, there was a significant correlation between the completeness of the amniotic sheet and IUFD [13]. This finding might be explained by the expansion of the lower segment in the second half of the pregnancy, eventually causing more tension on the adhesion and the surrounding fetal membranes.

According to the literature, ultrasonography, specifically three-dimensional transvaginal ultrasound (3D-TVUS), has shown high accuracy in the detection of IUA through interactive visualization and multiplanar reformatting [16]. Furthermore, a systematic review and meta-analysis of 20 studies investigating the prenatal sonographic detection of abnormal placentation reported a sensitivity of 87% and a specificity of 98% [17]. MRI is frequently used for confirmation [18]. In order to achieve a high level of accuracy, it is recommended that the findings of both imaging modalities be assessed concurrently [17].

The prevention of IUA can be achieved through the avoidance of forced or repeated intrauterine interventions, for example, by favoring medically induced abortions rather than curettage [19]. Moreover, the use of intrauterine contraceptive devices after resection of an IUA has been shown to decrease the recurrence of IUA [8]. In cases of antenatal diagnosis, a hysteroscopy is considered the best intervention to remove IUA due to its less damaging effects [19]. Additionally, estrogen has proven beneficial as a treatment after hysteroscopic surgery for Asherman syndrome to prevent the formation and recurrence of adhesions [10].

As most studies are retrospective case series, the available literature is limited. However, it supports the suspicion of possible fatal complications if IUA is diagnosed. For this reason, it is important to consider prompt fetal monitoring, maternal surveillance, and early delivery, depending on the clinical situation. The evacuation of the uterine cavity following abortion or delivery should be conducted with careful consideration, coupled with meticulous ultrasonographic guidance, to assure favorable outcomes [20]. In cases where abnormal placentation is suspected, it is advisable to plan for a cesarean section with a team of experienced clinicians in order to minimize the risk of severe postpartum hemorrhage.

## Conclusions

In conclusion, recent publications illustrate an increasing incidence of pregnancy complications in the presence of intrauterine adhesions. In efforts to decrease pregnancy complications, there should be prompt feto-maternal surveillance. If intrauterine adhesions are diagnosed antenatally, surgical resection can be offered to reduce potentially fatal complications.
